# Editorial: Molecular insights into fatty liver disease: pathogenesis, progression, and therapeutic strategies

**DOI:** 10.3389/fphar.2026.1805125

**Published:** 2026-02-25

**Authors:** Haibo Dong, Wei Guo, Ruichao Yue, Yue Feng, Jose C. Fernandez-Checa

**Affiliations:** 1 Center for Translational Biomedical Research, University of North Carolina at Greensboro, North Carolina Research Campus, Kannapolis, NC, United States; 2 State Key Lab of Veterinary Public Health and Safety, College of Veterinary Medicine, China Agricultural University, Beijing, China; 3 College of Animal Sciences, Fujian Agriculture and Forestry University, Fuzhou, China; 4 Department of Molecular and Cellular Medicine, Instituto de Investigaciones Biomédicas de Barcelona, Consejo Superior de Investigaciones Científicas, Barcelona, Spain; 5 Liver Unit, Hospital Clinic I Provincial de Barcelona, Instituto de Investigaciones Biomédicas August Pi i Sunyer (IDIBAPS), Barcelona, Spain; 6 Center for the Study of Liver and Gastrointestinal Diseases (CIBERehd), Carlos III National Institute of Health, Madrid, Spain; 7 Department of Medicine, Keck School of Medicine, University of Southern California, Los Angeles, CA, United States

**Keywords:** ALD, fatty liver disease, immunometabolism, lipotoxicity, MASLD, MetALD, therapy

Fatty liver disease is no longer a niche hepatology problem but is increasingly recognized as a systemic metabolic–inflammatory disorder. Large-scale epidemiological analyses have estimated that metabolic dysfunction–associated steatotic liver disease (MASLD) affects over 25% of the general population and is tightly associated with obesity, insulin resistance, and type 2 diabetes (Younossi et al., 2023; Cusi et al., 2025). In parallel, alcohol-associated liver disease (ALD), spanning from steatosis to cirrhosis, remains one of the leading causes of liver-related deaths worldwide. Its development, in addition to its unique triggers related to alcohol metabolism, is often linked to a dangerous combination of metabolic dysfunction (Gao et al., 2025; Pan et al., 2025). These observations challenge traditional binary disease classifications and underscore the need for integrated pathogenic frameworks. At the mechanistic level, metabolic overload and alcohol exposure function as primary upstream stressors that initiate hepatocellular injury. Excess nutrient availability promotes hepatic lipid accumulation and associated lipotoxicity, alters fatty acid flux, and induces insulin resistance, while ethanol metabolism generates toxic intermediates (e.g., acetaldehyde) and oxidative stress (Cheng et al., 2016; Lee et al., 2025). These primary hepatocellular stresses subsequently engage innate immune sensing and adaptive immune polarization, shaping whether injury resolves or progresses toward fibrogenesis. Importantly, innate and adaptive immune remodeling is increasingly recognized predominantly not as the initiating event but as a secondary amplification system that sustains inflammation and fibrogenesis once hepatocyte injury is established (Peiseler et al., 2022). This hierarchical organization helps explain why distinct etiologies converge on overlapping inflammatory and fibrotic phenotypes during disease progression.

At the cellular execution layer, while distinct upstream injuries are not entirely identical, they functionally converge on a limited set of conserved stress-response pathways. Lipotoxicity-induced organelle dysfunction, mitochondrial impairment, oxidative injury, and dysregulated wound-healing responses are common molecular nodes in both metabolic liver diseases and alcoholic liver diseases (Garcia-Ruiz and Fernandez-Checa, 2018; Kim and Kim, 2020; Prasun et al., 2021; Contreras-Zentella et al., 2025). These processes shape hepatocyte fate decisions, inflammatory signaling, and extracellular matrix remodeling, thereby providing a mechanistic basis for the overlapping histological features observed in MASLD and ALD, particularly during the transition from steatosis to steatohepatitis and fibrosis.

The rapid conceptual evolution of the field is reflected in recent changes to disease nomenclature. A multisociety Delphi consensus introduced steatotic liver disease as an umbrella term and repositioned NAFLD/NASH as MASLD/MASH, while explicitly defining overlap categories such as metabolic and alcohol related/associated liver disease (MetALD) (Rinella et al., 2023). This nomenclature update coincides with, but is conceptually distinct from, ongoing discussions comparing MASLD and MAFLD, in which MAFLD has been reported to better capture all-cause mortality as well as hepatic and extrahepatic outcomes that closely track fibrosis severity (Lonardo et al., 2025). Importantly, these parallel frameworks converge on a shared conclusion: metabolic dysfunction and alcohol exposure frequently coexist and interact. Consistent with this view, cohort studies further suggest that mixed metabolic–alcoholic phenotypes exhibit distinct trajectories of fibrosis progression and clinical outcomes, reinforcing the clinical relevance of overlap states (Sogabe et al., 2023; Marti-Aguado et al., 2024).

Within this evolving scenario, immunometabolic coupling has emerged as a central organizing principle. Hepatic immune cells respond dynamically to metabolic and toxic stress, with macrophages, dendritic cells, and lymphocyte subsets adopting context-dependent phenotypes that can either exacerbate injury or promote resolution. Reviews within this Research Topic emphasize that cytokine signaling networks integrate metabolic cues with inflammatory responses. Liu et al. synthesized evidence indicating that members of the interleukin-6 cytokine family participate in regulating lipid metabolism, insulin sensitivity, inflammatory amplification, and fibrogenesis in MASLD, supporting the view that cytokine pathways function as active immunometabolic regulators rather than passive downstream markers.

Beyond immune signaling, regulated inflammatory cell death pathways have gained increasing attention as critical links between hepatocyte injury and immune activation. Pyroptosis and related execution programs promote the release of danger-associated molecular patterns, thereby reinforcing inflammatory feedback loops (Farrell et al., 2018). In this context, Deng et al. provided evidence suggesting that short-term high-fat diet exposure synergizes with acute ethanol binge to exacerbate liver injury through oxidative stress, MAPK/NF-κB activation, and engagement of both canonical and non-canonical pyroptotic pathways. These findings provide mechanistic insight into the convergence of metabolic and alcohol-related stressors at the level of inflammatory execution programs, thereby offering a biologically grounded explanation for the heightened inflammatory injury observed in mixed-exposure contexts.

Translationally, the absence of effective pharmacological therapies for advanced fatty liver disease has long represented a major unmet need. This landscape is now rapidly changing. In 2024, the U.S. Food and Drug Administration approved resmetirom for adults with noncirrhotic MASH and moderate-to-advanced fibrosis, marking the first disease-modifying therapy for this indication (Huang et al., 2025). More recently, incretin-based therapies have demonstrated clinically meaningful histological improvements, reinforcing the therapeutic potential of targeting metabolic pathways upstream of inflammatory injury (Brouwers et al., 2024; Targher et al., 2025). These developments underscore the importance of mechanism-guided patient stratification and combination strategies.

Consistent with this direction, studies within this Research Topic highlight emerging therapeutic paradigms that integrate metabolic correction with immune modulation. González-Serrano et al. proposed that effective management of MASLD may require coordinated targeting of metabolic and immune pathways, rather than isolated correction of metabolic parameters alone. Complementing this conceptual framework, Comella et al. reported findings indicating that the SGLT2 inhibitor empagliflozin ameliorated hepatic steatosis, inflammation, and fibrosis in a preclinical MASLD model, accompanied by improvements in mitochondrial function and macrophage polarization toward pro-resolving phenotypes. These observations support the broader notion that metabolic drugs may exert hepatoprotective effects by reshaping immunometabolic homeostasis.

In parallel, interest is growing in complementary strategies that target oxidative stress, inflammation, and gut–liver interactions. A systematic synthesis by Hao et al. reviewed preclinical evidence suggesting that ginsenosides modulate inflammatory signaling, oxidative stress, insulin sensitivity, and gut microbiota composition in fatty liver disease models. While these findings remain largely preclinical and heterogeneous in methodological quality, they illustrate the expanding search for multi-target interventions capable of addressing the complex pathophysiology of MASLD and ALD.

Importantly, molecular insights alone are insufficient to translate mechanistic advances into clinical benefit without parallel progress in diagnostic stratification and implementation frameworks. Although liver biopsy remains the reference standard for diagnosing and staging steatohepatitis and fibrosis, its invasiveness, sampling variability, and limited scalability severely constrain its use for population-level screening and early disease interception (Ratziu et al., 2005; Davison et al., 2020; Nielsen et al., 2022). Consequently, fatty liver disease often follows a clinically silent course, with a substantial proportion of patients first identified at advanced stages characterized by established fibrosis or cirrhosis, where therapeutic reversibility is markedly diminished (Angulo et al., 2015; Dulai et al., 2017). This diagnostic delay reflects not only the insidious biology of disease progression but also the suboptimal sensitivity and specificity of commonly used non-invasive markers—such as serum transaminases and simple fibrosis scores—for detecting steatohepatitis and early fibrotic remodeling (Verma et al., 2013; Younossi et al., 2018). Population-level analyses further indicate that underdiagnosis is disproportionately pronounced in regions with high metabolic burden and limited access to advanced imaging or histological assessment, where reliance on basic anthropometric or biochemical indices fails to capture clinically meaningful disease heterogeneity (Paruk et al., 2019; Nielsen et al., 2022). In this regard, Olaez-Ramos et al. highlighted how epidemiological patterns and diagnostic constraints in Latin America shape disease recognition and outcomes, emphasizing that scalable, non-invasive risk stratification tools are essential for translating molecular advances into equitable clinical benefit.

Collectively, the studies assembled in this Research Topic reinforce a unifying view of fatty liver disease as a multifactorial, immunometabolic disorder shaped by metabolic stress, immune regulation, environmental exposure, and population-level determinants. Rather than emphasizing isolated pathways, these contributions converge on the concept that disease progression reflects the integration of conserved cellular stress responses with context-specific modifiers, including alcohol exposure, cytokine milieu, and therapeutic intervention.

In conclusion, the field of fatty liver disease is transitioning from descriptive expansion to intervention-ready precision. The emergence of mechanistically informed nomenclature, actionable therapeutic targets, and disease-modifying drugs drives a new phase in which molecular insights must be coupled with rigorous stratification and implementation strategies. By integrating mechanistic, translational, and epidemiological perspectives, this Research Topic provides a framework for understanding fatty liver disease as a systems-level disorder and for guiding future research toward durable clinical impact.
